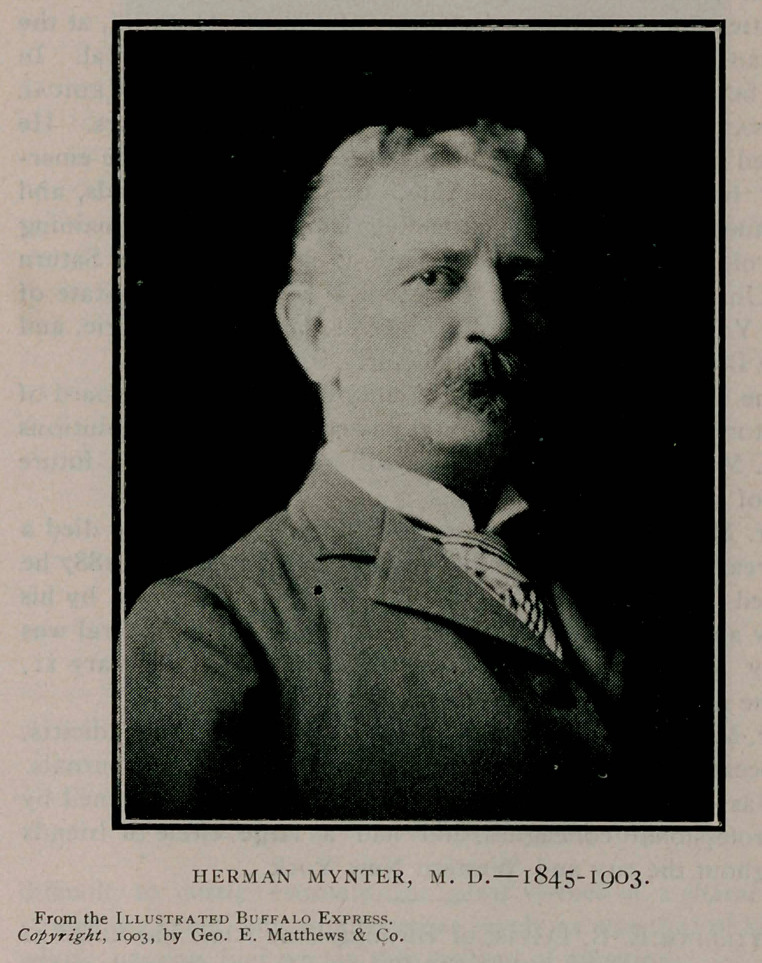# Dr. Herman Mynter

**Published:** 1903-03

**Authors:** 


### OBITUARY.

Dr. Herman Mynter, of Buffalo, died at his home, 466 Delaware Avenue, February 9, 1903, of arterio-sclerosis, aged 57 years. His last illness was about two weeks in duration, though he had suffered more or less for nearly eighteen months. He made a tour in Europe during the summer of 1902, which

HERMAN MYNTER, M. D.—1845-1903.

From the Illustrated Buffalo Express.
*Copyright*, 1903, by Geo. E. Matthews & Co.

seemed to benefit him, and on his return he resumed and continued at his professional work until within a fortnight of his death.

Herman Mynter was born in Karebaek, Denmark, took the university degrees at Copenhagen in 1871, served in the medical corps of the Danish navy about two years, and in the army one year, in compliance with the laws of his native land. He came to America in 1875, and immediately located in Buffalo, where he continued to practise his profession until his last illness.

Dr. Mynter was a surgeon of repute, and was appointed professor of surgery in Niagara University when the institution was organised. After that medical school was discontinued he became clinical professor of surgery at the University of Buffalo. He was attending surgeon at the Sisters of Charity Hospital, at the German Deaconess's Home, and at the German Hospital. In 1879 he became one of the owners of the BUFFALO MEDICAL JOURNAL, and was one of the editors for a few years. He assisted at the operation upon President McKinley at the emergency hospital on the Pan-American Exposition grounds, and continued as one of the attending staff during the remaining days of the President's life. He was a member of the Saturn and University Clubs, of the Medical Society of the State of New York, of the Medical Society of the County of Erie, and of the Buffalo Academy of Medicine.

The Medical Society of the County of Erie and the Board of Directors of the German Hospital passed appropriate resolutions in Dr. Mynter's memory. These will be published in a future issue of the JOURNAL.

Dr. Mynter was twice married, his first wife having died a few years after marriage, leaving two daughters. In 1887 he married Miss Harriet Martin Buell. He is survived by his widow and two daughters, Agnes and Emily. His funeral was largely attended from his home on Wednesday, February 11, and the interment was at Forest Lawn.

Dr. Mynter was the author of a monograph on Appendicitis, and occasionally contributed articles to this and other journals. He was a man of strong individuality, was well esteemed by his professional colleagues, and had a large circle of friends throughout the city and Western New York.

## Figures and Tables

**Figure f1:**